# Late-Onset Running Biphasically Improves Redox Balance, Energy- and Methylglyoxal-Related Status, as well as SIRT1 Expression in Mouse Hippocampus

**DOI:** 10.1371/journal.pone.0048334

**Published:** 2012-10-26

**Authors:** Stefano Falone, Antonella D'Alessandro, Alessandro Mirabilio, Marisa Cacchio, Carmine Di Ilio, Silvia Di Loreto, Fernanda Amicarelli

**Affiliations:** 1 Department of Basic and Applied Biology, University of L'Aquila, L'Aquila (AQ), Italy; 2 Department of Basic and Applied Medical Sciences, University “G. d'Annunzio”, Chieti Scalo (CH), Italy; 3 Department of Biomedical Sciences, University “G. d'Annunzio”, Chieti Scalo (CH), Italy; 4 Institute of Translational Pharmacology (IFT) – National Research Council (CNR), L'Aquila (AQ), Italy; Oregon Health & Science University, United States of America

## Abstract

Despite the active research in this field, molecular mechanisms underlying exercise-induced beneficial effects on brain physiology and functions are still matter of debate, especially with regard to biological processes activated by regular exercise affecting the onset and progression of hippocampal aging in individuals unfamiliar with habitual physical activity. Since such responses seem to be mediated by changes in antioxidative, antiglycative and metabolic status, a possible exercise-induced coordinated response involving redox, methylglyoxal- and sirtuin-related molecular networks may be hypothesized. In this study, hippocampi of CD1 mice undergoing the transition from mature to middle age were analyzed for redox-related profile, oxidative and methylglyoxal-dependent damage patterns, energy metabolism, sirtuin1 and glyoxalase1 expression after a 2- or 4-mo treadmill running program. Our findings suggested that the 4-mo regular running lowered the chance of dicarbonyl and oxidative stress, activated mitochondrial catabolism and preserved sirtuin1-related neuroprotection. Surprisingly, the same cellular pathways were negatively affected by the first 2 months of exercise, thus showing an interesting biphasic response. In conclusion, the duration of exercise caused a profound shift in the response to regular running within the rodent hippocampus in a time-dependent fashion. This research revealed important details of the interaction between exercise and mammal hippocampus during the transition from mature to middle age, and this might help to develop non-pharmacological approaches aimed at retarding brain senescence, even in individuals unfamiliar with habitual exercise.

## Introduction

Reactive oxygen species (ROS) and accumulation of chemically-damaged biomolecules, together with impaired scavenging capacities, are known to play crucial roles in the onset and progression of cellular senescence as well as in many age-associated dysfunctions [1]–[3].

Since the central nervous system (CNS) is extremely prone to the pro-oxidant damage, mainly due to several physiological and biomolecular properties of CNS, brain activities and functions are severely impaired by age-associated pro-oxidant challenges [4]–[6].

Although exercise promotes ROS overproduction [7], [8], regular and moderate exercise paradigms improve psycho-physical functions, retards aging and lowers the risk of developing many age-related diseases, as well as it seems to reduce the age-dependent cognitive decline, preserving learning and memory [9]–[14]. This apparent paradox may be explained by taking into account the hormetic responses triggered by habitual exercise through the induction of molecular adaptations, such as the activation of major antioxidant enzymatic systems (e.g., superoxide dismutase, SOD, and catalase, CAT) [15]–[17].

The oxidized form of nicotinamide adenine dinucleotide (NAD^+^) is a pleiotropic factor which probably mediates some of the effects of exercise in the CNS. NAD^+^ is a metabolic energy sensor which is strictly linked to cellular redox-related functions, beyond being essential to the activity the NAD-dependent deacetylase sirtuin-1 (SIRT1), one of the most important determinants of longevity [18]–[25], and to the activity of glyceraldehyde 3-phosphate dehydrogenase (GAPDH), a crucial enzyme which removes the glycolytic precursors of the methylglyoxal (MG). MG is a very reactive pro-oxidant α-ketoaldehyde, which is mainly responsible for the most damaging age-related glycating reactions in vivo; in particular, MG can modify proteins by producing advanced glycation end products (AGEs) and protein adducts, such as the arg-pyrimidine [26]–[30]. MG can damage cellular components due to either increased levels of its precursors or a reduced efficiency of the main route through which MG is removed (e.g., the glyoxalases 1 and 2, GLO1 and GLO2) [31]–[38]. Despite the active research in this field, no previous work analyzed the exercise-dependent effects on the expression of the MG-related detoxification system within the mouse hippocampus. In addition, though some authors reported that long-term running stimulates SIRT1 activation and mitochondria efficiency in non-nervous tissues [39], only few authors attempted to extend this studies to the mammal CNS [40]. Such an information may be important as interventions aimed at preserving the mitochondrial efficiency (e.g., the caloric restriction) seem to retard signifcantly the overall senescence process in many tissues and organisms [23]–[25].

**Figure 1:**
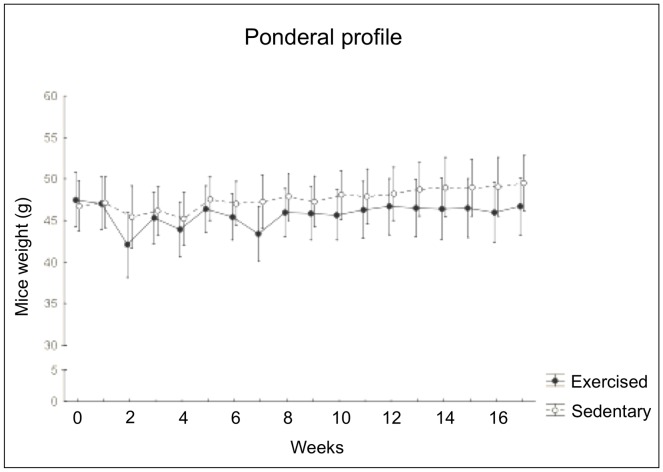
Ponderal profiles of mice undergoing a long-term moderate treadmill running. No significant age-dependent main effect was detected on body weight, neither did treadmill running induce significant alterations in ponderal state. Values were given as means ± std. dev.

The present study was aimed at filling existing knowledge gaps, by verifying in a time-dependent manner whether a moderate and regular exercise regimen initiated in mature age, a biological period in which cognitive abilities are known to start declining [41], could perturb the interplay among the oxidative damage pattern, the redox-, energy- and MG-related profiles in hippocampal formations of mature mice. Our aim was also to detect possible changes elicited by the long-term enforced exercise regimen in the SIRT1 expression pattern. Time-course analysis was suggested by some findings reporting that in muscle tissues the duration of exercise regimen could be critical in triggering specific biological responses [15], [16], [42]–[46].

**Figure 2:**
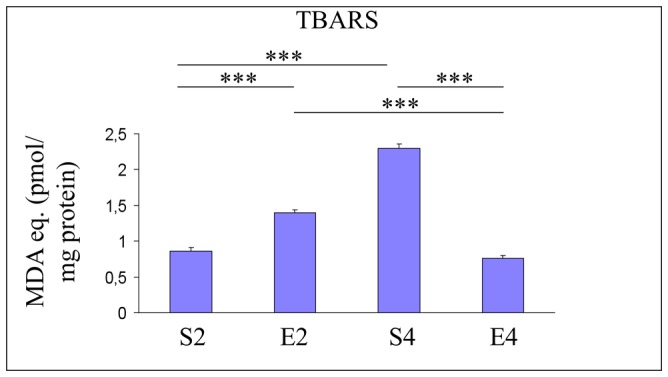
Hippocampal peroxidative damage in mice undergoing 2- or 4-mo moderate treadmill running. Assessment of hippocampal levels of thiobarbituric acid-reactive substances (TBARS) levels in mature CD-1 female mice undergoing a two- or four-month moderate and regular treadmill-based exercise program (E2 or E4, respectively); age-matched sedentary animals (S2, S4) were used as controls (n = 12 per group). TBARS levels were found to be increased by the sedentary aging (S4 vs S2). 2-mo exercise increased TBARS hippocampal concentrations (E2 vs S2), while 4-mo running decreased TBARS levels (E4 vs S4). Values were given as means ± std. dev. The level of statistical significance was computed by using two-way ANOVA and post-hoc Newman-Keuls test: *** P<0.001; Experiments were performed in triplicate.

Our findings extended the knowledge on how a lately-initiated regular and moderate running program modulates the onset of critical hallmarks of cellular senescence during the transition from adult to middle age within the mammal CNS.

**Figure 3:**
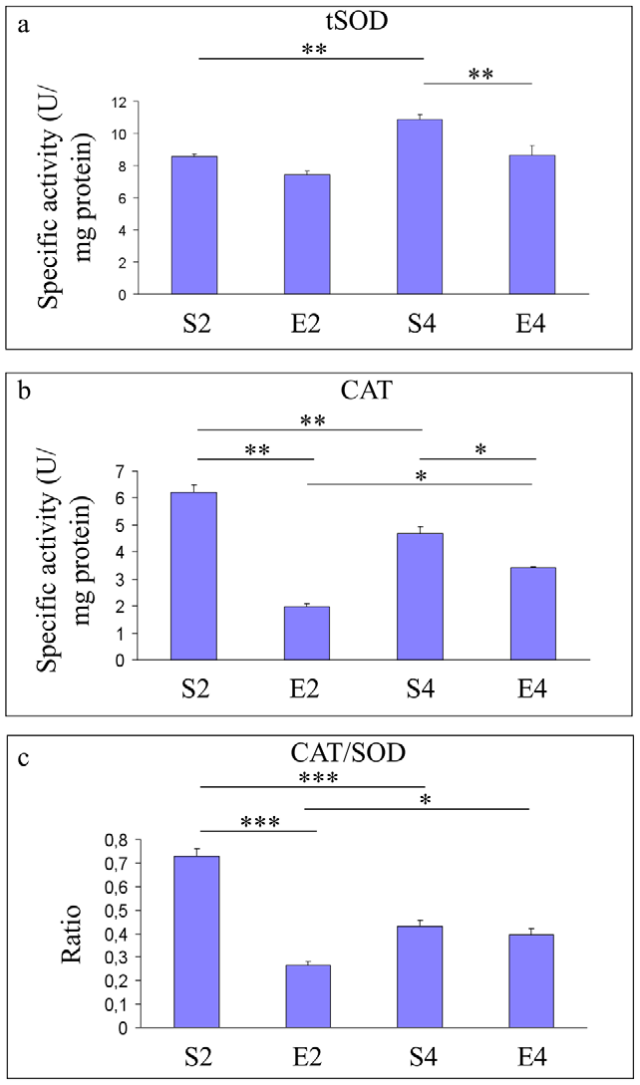
Antioxidant enzymatic defense in hippocampi of mice undergoing 2- or 4-mo moderate treadmill running. Evaluation of hippocampal specific activities of the major antioxidant enzymes total superoxide dismutase (tSOD) and catalase (CAT) in mature CD-1 female mice undergoing a two- or four-month moderate and regular treadmill-based exercise program (E2 or E4, respectively); age-matched sedentary animals (S2, S4) were used as controls (n = 12 per group). Sedentary aging increased the tSOD activity (S4 vs S2, panel a), but decreased both the CAT activity (S4 vs S2, panel b) (panel b) and the CAT/tSOD ratio (S4 vs S2) (panel c). 2-mo running reduced the CAT specific activity (E2 vs S2) and the CAT/tSOD ratio (E2 vs S2) (panels b and c, respectively), while 2-mo exercise did not change the tSOD activity (E2 vs S2) (panel a). 4-mo running decreased specific activities of both tSOD and CAT (E4 vs S4) (panels a and b, respectively), without changing the CAT/tSOD ratio (panel c). Values were given as means ± std. dev. The level of statistical significance was computed by using two-way ANOVA and post-hoc Newman-Keuls test: *** P<0.001; ** P<0.01; * P<0.05. Experiments were performed in triplicate.

## Materials and Methods

### Chemicals and antibodies

The monoclonal antibody against the arg-pyrimidine adduct was purchased from Cosmo Bio Co., LTD (cat. NOF-N213430-EX; Tokio, Japan). The rabbit anti-glyoxalase I (cat. sc-67351) antibody was supplied by Santa Cruz Biotecnology, Inc. (Santa Cruz, CA, USA). Rabbit anti-SIRT1 (cat. ab75435) and anti-β-actin (cat. ab8227) antibodies were purchased from Abcam (Cambridge, UK). Acrylamide/bis acrylamide (cat. 1610125), premixed Laemmli sample buffer (cat. 1610737), Kaleidoscope prestained standards (cat. 1610324), blotting grade blocker (cat. 1706404) and Immun-Blot PVDF Sandwiches (cat. 1620239) were purchased from Bio-Rad Laboratories (Hercules, CA, USA). Thermo Fisher Scientific (Rockford, IL, USA) provided the metal-enhanced 3′,3-diaminobenzidine (DAB) substrate kit (cat. 34065) and the BCA protein assay kit (cat. 23225). Cayman Chemical (Ann Arbor, MI, USA) supplied the thiobarbituric acid reactive substances (TBARS) (cat. 10009055) and the glutathione (cat. 703002) assay kits. The EnzyChrom NAD^+^/NADH assay kit (cat. ECND-100) was supplied by BioAssay Systems (Hayward, CA, USA). If not otherwise specified, all chemicals and substrates used in Western immunoblots, spectrophotometrical assessments and microplate-based measurements were supplied by Sigma Aldrich (St. Louis, MO, USA).

**Figure 4:**
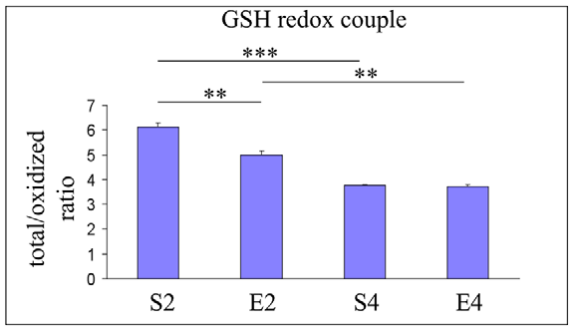
Glutathione redox status in hippocampi of mice undergoing 2- or 4-mo moderate treadmill running. Evaluation of the glutathione redox balance in hippocampi of mature CD1 female mice undergoing a two- or four-month moderate and regular treadmill-based exercise program (E2 or E4, respectively); age-matched sedentary animals (S2, S4) were used as controls (n = 12 per group). A significant age-dependent decrease in the total vs oxidized glutathione ratio was found within mouse hippocampal formations (S4 vs S2). 2-mo running decreased the total vs disulfide glutathione ratio (E2 vs S2), whereas no significant change was induced by 4-mo exercise (E4 vs S4). Values were given as means ± std. dev. The level of statistical significance was computed by using two-way ANOVA and post-hoc Newman-Keuls test: *** P<0.001; ** P<0.01. Experiments were performed in triplicate.

### Animals and running protocol

9-month old CD1 female mice (N = 48; Harlan Laboratories, Inc., Frederick, MD, USA) were used in this research. All subjects (45–50 grams) were acclimatized to housing conditions for 10 days, in the animal laboratories of the Excellence Research Center on Aging of the University Foundation “G. d'Annunzio” of Chieti, Italy (22±2°C, 12–12 h light-dark cycle with lights on from 8 a.m. to 8 p.m., free access to water and food, six animals per cage).

**Figure 5:**
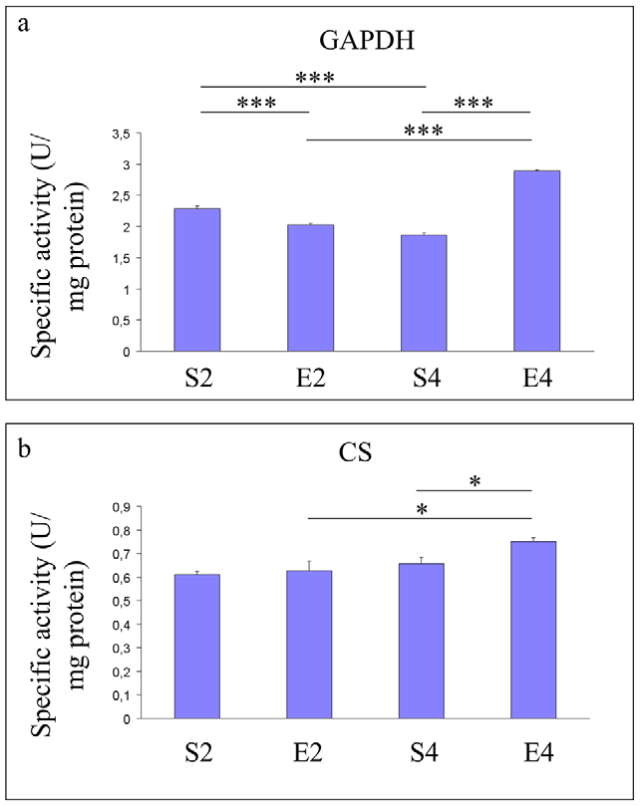
Energy-related enzymatic metabolism in hippocampi of mice undergoing 2- or 4-mo moderate treadmill running. Assessment of hippocampal specific activities of glyceraldehydes-3-phosphate dehydrogenase (GAPDH) and citrate synthase (CS) in mature CD-1 female mice undergoing a two- or four-month moderate and regular treadmill-based exercise program (E2 or E4, respectively); age-matched sedentary animals (S2, S4) were used as controls (n = 12 per group). An age-dependent decrease in GAPDH specific activity (S4 vs S2) (panel a) was detected, whereas no significant age-related changes in CS activity were observed (S4 vs S2) (panel b). 2-mo running reduced the GAPDH specific activity (E2 vs S2) (panel a), yet 2-mo exercise did not alter the CS activity (E2 vs S2) (panel b). 4-mo running increased activities of both GAPDH and CS (E4 vs S4) (panels a and b, respectively). Values were given as means ± std. dev. The level of statistical significance was computed by using two-way ANOVA and post-hoc Newman-Keuls test: *** P<0.001; * P<0.05. Experiments were performed in triplicate.

In this research, we used a forced treadmill-based running program, which is a standard form of aerobic exercise often used for experiments on rodents, as some authors argued that an enforced exercise protocol is more easily controlled and less likely biased depending on different genetic backgrounds [47]–[49].

**Figure 6:**
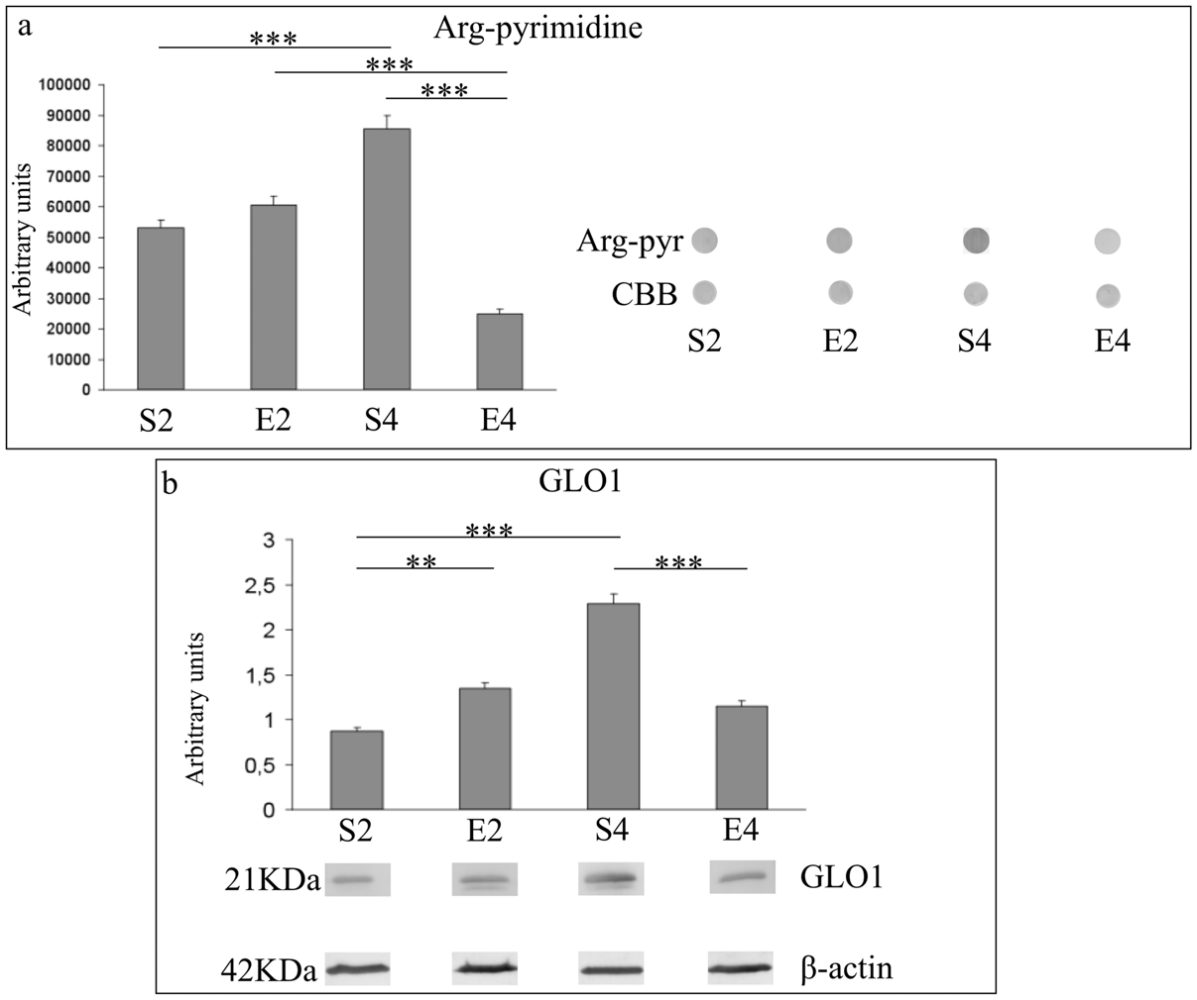
MG-related protein damage and expression of MG-targeting detoxification system in hippocampi of mice undergoing 2- or 4-mo moderate treadmill running. Immunoreactivity levels against arg-pyrimidine and protein expression of glyoxalase 1 (GLO1) in hippocampi of mature CD-1 female mice undergoing a two- or four-month moderate and regular treadmill-based exercise program (E2 or E4, respectively); age-matched sedentary animals (S2, S4) were used as controls (n = 12 per group). As shown in panel a, an age-dependent increase in the levels of MG-damaged protein was detected (S4 vs S2), and an increasing trend in MG-related protein damage was observed after 2 months of running (E2 vs S2). The immunoreactivity against arg-pyrimidine decreased after 4 months of regular exercise (E4 vs S4). Dot-blots signals were normalized against the Comassie Brilliant Blue-based total protein staining. In the panel a, right section, representative dot immunoblots of three independent experiments were reported. As reported in panel b, an age-dependent increase in the GLO1 protein expression levels was detected (S4 vs S2). 2-mo exercise increased GLO1 protein levels (E2 vs S2), while the expression levels of GLO1 decreased after four months of regular running (E4 vs S4). Immunosignals were normalized against the housekeeping β-actin. Representative western immunoblots of three independent experiments were reported in panel b. Values were given as means ± std. dev. The level of statistical significance was computed by using two-way ANOVA and post-hoc Newman-Keuls test: *** P<0.001; ** P<0.01. Experiments were performed in triplicate.

All animals were familiarized to the Exer 3/6 motorized low-noise treadmill (Columbus Instruments, Columbus, OH, USA) for 2 weeks (9 m/min for 10 min, 5 days/week).

**Figure 7:**
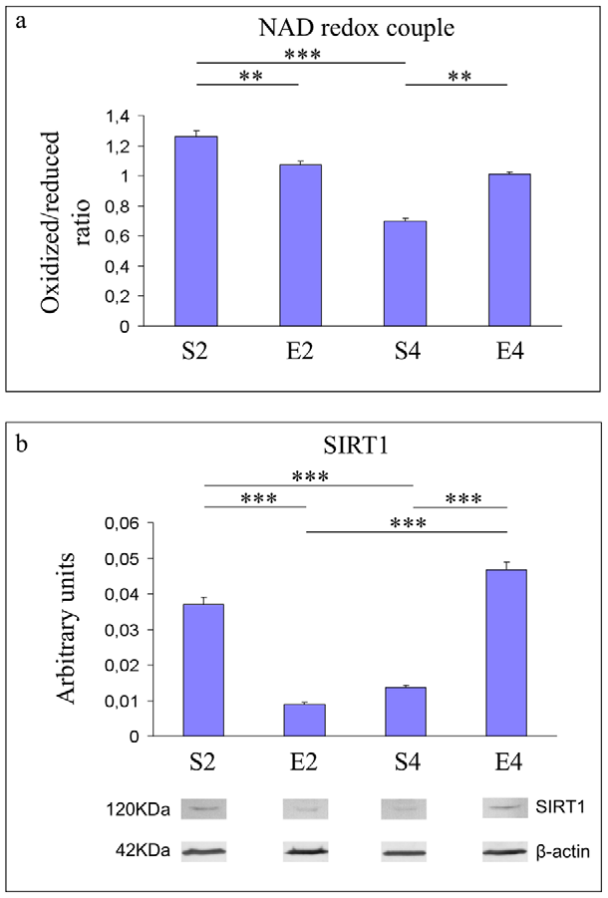
NAD redox balance and expression of SIRT1 in hippocampi of mice undergoing 2- or 4-mo moderate treadmill running. Assessment of nicotinamide adenine dinucleotide redox balance (panel a) and Western immunoblot against SIRT1 (panel b) in hippocampi of mature CD-1 female mice undergoing a two- or four-month moderate and regular treadmill-based exercise program (E2 or E4, respectively); age-matched sedentary animals (S2, S4) were used as controls (n = 12 per group). Both the NAD redox ratio and the SIRT1 protein level were decreased in an age-dependent fashion (S4 vs S2) (panels a and b, respectively). 2-mo exercise reduced both the NAD^+^/NADH ratio and the expression level of SIRT1 (E2 vs S2) (panels a and b, respectively), whereas both the NAD redox balance and the protein level of SIRT1 increased after 4-mo exercise (E4 vs S4) (panels a and b, respectively). Western blot signals were normalized against the housekeeping β-actin. Representative western immunoblots of three independent experiments were reported in panel b. Values were given as means ± std. dev. The level of statistical significance was computed by using two-way ANOVA and post-hoc Newman-Keuls test: *** P<0.001; ** P<0.01. Experiments were performed in triplicate.

As described previously [50], animals were randomly divided into four groups (12 mice each): subjects committed to be sacrificed 2 or 4 months later, in presence (E2, E4) or absence (S2, S4) of physical exercise protocol. Exercised groups started the training program (3 min warm-up at 5 m/min, 20 min at 13 m/min, 3 min cool-down at 5 m/min, zero inclination), 5 days/week. The running work load reached the final time of 20 min/day with incremental steps of 1 min/day, starting from 10 min/day. Mice were motivated to run by gentle hand prodding. Sedentary groups were exposed at the same environmental condition (handling, treadmill motor noise, vibration and deprivation of food and water) while exercised animals performed their daily running sessions. Food consumption and animal weight were daily monitored throughout the experiment. 24 h after the last training session, mice were rapidly sacrificed by decapitation and hippocampal formations were surgically removed and stored at −80°C until further analysis. Every possible effort was taken in order to minimize both the number and the suffering of used animals, in accordance with principles of the EC Directive 86/609/EEC for animal experiments. Formal approval for experimental procedures was provided by the Ministry of Health (protocol 13/97–A).

### Sample preparation for enzymatic activity

Hippocampal formations were homogenized by Ultra-Turrax in 5 vol of 0.1M phosphate buffer (pH 7), containing 1 mM EDTA and 1.5 mM DTT, for subsequent analyses of GAPDH, or 0.1% Triton X-100, for SOD, CAT and CS. Then, samples were sonicated (cycle 0.5 – amplitude 50%) three times and centrifuged at 15,000 g for 30 min at 4°C. Extracts were used for spectrophotometric assessments of enzymatic activities and protein content [51].

### Superoxide dismutase (SOD)

Total SOD (EC 1.15.1.1) activity was assayed by its ability to inhibit the autoxidation of epinephrine, which was monitored spectrophotometrically at 480 nm at 30°C. An appropriate amount of the extract was used to obtain about 50% inhibition of the epinefrine autoxidation in a 50 mM NaHCO_3_ – 0.1 mM EDTA buffer (pH 10.2). One unit of SOD activity was assumed to provide 50% of inhibition [52].

### Catalase (CAT)

CAT (EC 1.11.1.6) activity was assayed in a 50 mM KH_2_PO_4_ buffer (pH 6.8) by monitoring the decomposition of 10 mM H_2_O_2_ at 240 nm (ε = 0.04 mmol^−1^dm^−3^cm^−1^) [53]. One unit of enzyme activity was defined as 1 μmol of H_2_O_2_ reduced/min at 25°C.

### Glyceraldehyde-3-phosphate dehydrogenase (GAPDH)

GAPDH (EC 1.2.1.12) activity was assayed in a 94 mM Tris buffer (pH 7.6) supplemented with 1 mM EDTA and 2 mM MgSO_4_. The enzyme assay mixture contained also 1.2 mM ATP, 0.23 mM NADH, 20 mU/μl phosphoglycerate kinase (PGK) and 14.3 mM glycerate 3-P [54]. NADH oxidation was followed at 340 nm and one unit of enzyme activity was defined as 1 μmol NADH oxidized/min at 25°C.

### Citrate synthase (CS)

CS (EC 4.1.3.7) activity was assayed in a 72 mM Tris-HCl buffer (pH 8.1), according to the method described by Srere [55]. The reaction was monitored spectrophotometrically by starting the reaction between acetyl-CoA (0.15 mM) and oxaloacetate (0.5 mM), and coupling the release of free CoA-SH to the conversion of a colorimetric reagent, the 5,5-dithiobis-(2-nitrobenzoic acid) (DTNB, 0.1 mM) into the 5-thio-2-nitrobenzoic acid (TNB). The reaction was monitored at 412 nm and one unit of enzyme activity was defined as 1 μmol citrate produced/min at 25°C.

### Thiobarbituric acid-reactive substances (TBARS)

The measurement of TBARS is a well-established used to detect lipid peroxidative molecular damage through the photometric detection of the thiobarbituric acid-conjugated adduct [56]. In brief, 100 μl of RIPA-homogenized hippocampal formations were added in triplicate to 100 μl of SDS and 4 ml of Colour Reagent, as suggested by the manufacturer. Then, the reaction mixtures were incubated for one hour in boiling water and centrifuged at 1,600 g for 10 min at 4°C. Supernatants were read at 532 nm by a Lamba25 spectrophotometer (PerkinElmer Inc., Waltham, MA, USA). A linear calibration curve was computed from pure malondialdehyde (MDA)-containing reactions (range: 0–50 μM).

### Glutathione redox couple ratio

Total (tGSH) and oxidized glutathione (GSSG) levels were measured through the enzymatic recycling method described by Baker and colleagues [57], exploiting the reaction of the sulfhydril group of GSH with 5,5′-dithiobis-2-nitrobenzoic acid (DTNB, Ellman's reagent) and the subsequent formation of the yellow-colored 5-thio-2-nitrobenzoic acid (TNB). Quantification of GSSG was accomplished by first derivatizing the reduced glutathione with 2-vinylpyridine, as recommended by the manufacturer. In brief, hippocampal formations were homogenized in 5 vol of 100 mM KH_2_PO_4_ buffer, 1 mM EDTA (pH 7) and then centrifuged at 10,000 g for 15 min at 4°C. Supernatants were collected, deproteinized with 5% (w/v) metaphosphoric acid and centrifuged at 4,000 g for 5 min. 50 μl of protein-free supernatants were added in triplicate to 150 μl of Assay Cocktail, as suggested by the manufacturer. The absorbance at 405 nm was monitored by a Victor3 microplate reader (PerkinElmer) for 30 min, with intervals of 5 min each. A linear calibration curve was obtained from pure GSSG-and GSH-containing reactions (range: 0–8 μM GSSG, 0–16 μM tGSH).

### Nicotinamide adenine dinucleotide (NAD) redox couple ratio

NAD^+^ to NADH ratio was measured through an alcohol dehydrogenase cycling reaction, in which a tetrazolium dye (MTT) was reduced by NADH in the presence of phenazine methosulfate (PMS) [58]. The intensity of the reduced product color was proportionate to the NAD^+^ or NADH concentration in the sample. The determination of both NAD^+^ and NADH levels required extractions from two separate samples. Briefly, samples were homogenized with 5 vol of NAD^+^ or NADH extraction buffer and post-centrifugation supernatants were heated at 60°C for 5 min. Then, samples were neutralized with an 1 vol of the opposite extraction buffer and mixed with 20 μl of Assay Buffer, as recommended by the manufacturer. Samples were vortexed and centrifuged at 15,000 g for 5 min. The absorbance at 565 nm was detected by a Victor3 microplate reader (PerkinElmer) for 30 min, with intervals of 5 min each. A linear calibration curve was computed from pure NAD^+^-containing reactions (range: 0–10 μM).

### Western immunoblot

Hippocampal formations were homogenized in 5 vol of RIPA buffer supplemented with 1% protease inhibitor cocktail and 2% phosphatase inhibitor cocktails I/II and sonicated (cycle 0.5-amplitude 50%). Samples were, then, centrifuged at 15,000g for 30 min at 4°C and total protein content was assessed by bicinchoninic acid (BCA) method, using bovine serum albumin (BSA) as the standard [59]. Denaturated samples (25 μg total proteins) were run through polyacrylamide denaturing gels (12–15%) and bands were transferred onto methanol-activated polyvinylidene fluoride (PVDF) sheets by wet electrophoretic transfer [60], [61]. Non-specific binding sites were blocked with 5% (w/v) non-fat dry milk in Tris-buffered saline containing 0.05% (v/v) Tween 20 (TBS–T) for 1 h. Then, membranes were incubated with TBS-T containing primary rabbit polyclonal antibodies overnight at 4°C, with the following dilution conditions: anti-GLO1 (1∶400), anti-SIRT1 (1∶200), anti-β-actin (1∶1000). PVDF sheets were washed 3 times with TBS-T (5 min each) and incubated with a horseradish peroxidase (HRP)-conjugated secondary anti-rabbit antibody (dil. 1∶2,000) for 2 h. Membranes were washed 3 times with TBS (5 min each) and the specific immune complexes were detected by using metal-enhanced DAB substrate kit, by following manufacturer's instructions. Band intensities were acquired by digital scanning and processed through Nonlinear Dynamics TotalLab software. Protein of interest-related data were normalized to the signals relative to the β-actin housekeeping protein and given as relative units (RU). Experiments were performed in triplicate.

### Arg-pyrimidine-directed dot immunoblot

Twenty-five micrograms of RIPA-extracted proteins (see Western immunoblot, sub-section) were spotted onto methanol-activated PVDF sheets and air dried for 10 min. Membranes were blocked with non-fat dry milk for 1 h and incubated overnight at 4∞C with TBS-T containing the monoclonal antibody anti-arg-pyrimidine (dil. 1∶100). Sheets were washed 3 times with TBS-T (5 min each) and incubated with TBS-T containing the horseradish peroxidase-conjugated secondary antibody (dil. 1∶8000) for 2 h. Membranes were washed 3 times with TBS (5 min each) and immune complexes were detected by using the metal-enhanced DAB substrate kit, as recommended by the supplier. Membranes were digitally acquired and data analysis was performed after normalization versus Brilliant Blue Coomassie-R-stained total proteins, by using ImageJ and Nonlinear Dynamics TotalLab softwares [50], [62]. Results were given as relative units (RU). The experiments were performed in triplicate.

### Statistical analysis

Results were given as means ± standard deviations. Statistical analyses were performed by using Statsoft Statistica 7 and SyStat SigmaStat v3.5 softwares. Ponderal profiles were analyzed by two-way ANOVA for repeated measures. Two-way ANOVA was applied in order to detect significant main effects due to time, life style and interactions between the main factors. When appropriate, post-hoc Newman-Keuls tests for multiple comparisons were used. The null hypothesis was rejected when P<0.05.

## Results

### Effects of early senescence and long-term exercise on ponderal state and food intake

No significant main effect of physiological early senescence was detected on body weight (Fig. 1) or food consumption (not shown). Treadmill running induced no significant alterations in ponderal state (Fig. 1) or appetence behaviour (not shown).

### Age- and 2- or 4-mo exercise-dependent effects on oxidative damage and antioxidant/redox status

As shown in Fig. 2, an age-dependent elevation of TBARS concentrations was detected within mouse hippocampal formations (P<0.001, S4 vs S2). TBARS levels were increased by 2-mo exercise (P<0.001, E2 vs S2), while 4-mo running decreased hippocampal TBARS levels (P<0.001) (Fig. 2, E4 vs S4).

As shown in Fig. 3, we found an age-dependent increase of the tSOD specific activity (P<0.01, S4 vs S2) (panel a), together with a decreased CAT activity (P<0.01, S4 vs S2) (panel b) and a lower CAT/SOD ratio (P<0.001, S4 vs S2) (panel c). 2-mo running reduced the CAT specific activity (P<0.01, E2 vs S2) and the CAT/tSOD balance (P<0.001, E2 vs S2) (panels b and c, respectively), without affecting significantly the tSOD activity (Fig. 3a, E2 vs S2). 4-mo exercise decreased specific activities of both tSOD (P<0.01) and CAT (P<0.05) (Figs. 3a and b, E4 vs S4), without changing the CAT/tSOD ratio (Fig. 3c, E4 vs S4).

As shown in Fig. 4, we detected an age-dependent decrease in the total vs oxidized glutathione ratio (P<0.001, S4 vs S2). 2-mo running lowered the total vs disulfide glutathione ratio (P<0.01, E2 vs S2), whereas no exercise-dependent variation was observed after 4 months of running (E4 vs S4).

### Age- and 2- or 4-mo exercise-dependent effects on glyceraldehyde-3-phosphate dehydrogenase and citrate synthase enzymatic activities

As reported in Fig. 5, the GAPDH enzymatic activity decreased in an age-dependent fashion (P<0.001, S4 vs S2) (panel a), whereas no significant age-related changes in CS activity were observed (S4 vs S2) (panel b). 2-mo running reduced the hippocampal GAPDH specific activity (P<0.001, E2 vs S2) (Fig. 5a), without changing significantly the CS activity (Fig. 5b, E2 vs S2). 4-mo running increased the specific activities of both GAPDH (P<0.001) and CS (P<0.05) (Figs. 5a and b, respectively, E4 vs S4).

### Age- and 2- or 4-mo exercise-dependent effects on MG-related protein damage and expression of glyoxalase 1

As shown in Fig. 6 (panel a), an age-dependent increase of MG-damaged protein levels (P<0.001, S4 vs S2) was observed. 2-mo running slightly increased the MG-related immunosignal (S2 vs E2). Conversely, the level of MG-dependent protein damage was lowered by 4-mo regular treadmill running (P<0.001, S4 vs S2).

As reported in Fig. 6 (panel b), an age-dependent increase in the GLO1 protein expression was found (P<0.001; S4 vs S2). 2-mo exercise enhanced the protein expression of GLO1 (P<0.01, E2 vs S2). On the contrary, GLO1 espression was lowered by four months of habitual running (P<0.001, E4 vs S4).

### Age- and 2- or 4-mo exercise-dependent effects on the NAD redox balance and expression of SIRT1

As shown in Fig. 7, both the NAD redox balance (P<0.001) and the protein expression level of SIRT1 (P<0.001) were decreased in an age-dependent manner (S4 vs S2, panels a and b, respectively). 2-mo running reduced both the NAD^+^/NADH ratio (P<0.01) and the protein expression of SIRT1 (P<0.001) (E2 vs S2), whereas, NAD redox balance and SIRT1 protein levels were elevated after four months of running (P<0.01 and P<0.001, respectively, E4 vs S4).

## Discussion

Our results suggested that the four-month moderate running program, even when initiated lately in mice unfamiliar with exercise, was able to trigger multiple adaptive responses within the hippocampal formations of mice undergoing the transition from mature to middle age, a biological period in which we already showed profound biochemical and molecular changes occurring within mouse brain cortices [50]. The 4-mo running regimen strongly inhibited the age-dependent increase in hippocampal lipid peroxidative damage (Fig. 2). However, this protective effect was not achieved through the enhancement of SOD- and CAT- antioxidant defense systems or via an overall increase in reduced GSH equivalents, since we did not observe beneficial effects induced by 4-mo exercise on either the age-dependent reduction in hydrogen peroxide scavenging capacity (Fig. 3c) or the age-related drop of reduced GSH (Fig. 4). The decreased peroxidative damage, in absence of major ROS-targeting antioxidant responses, might be explained by considering that the 4-mo exercise regimen completely reverted both the age-dependent reduction in GAPDH activity and the age-associated collapse of the oxidized NAD concentrations (Figs. 5a and 7a, respectively). This biochemical pattern strongly suggested that in the time period investigated, a physiological decrease in the glycolytic flow of triose-phosphates occurred and this, in turn, could facilitate the endogenous overproduction of MG [29]. In agreement with this scenario, we detected an age-dependent increase in arg-pyrimidine derivatives within the hippocampi of unexercised mice, which was dramatically reverted by the 4-mo running program (Fig. 6a). These findings suggested that a lately-initiated regular exercise established a hippocampal biochemical environment in which no MG-related threat was occurred and no MG-targeting adaptive response was required. Coherently, the 4-mo exercise program inhibited the age-dependent elevation of GLO1 protein expression (Fig. 6b).

The four-month exercise program enhanced the aerobic utilization of energy precursors (Fig. 5b); the dependence on cellular NAD^+^ links the cellular metabolic state to SIRT1. With this regard, the 4-mo running regimen totally reverted the age-related decline of SIRT1 protein levels (Fig. 7b). This, together with the higher NAD^+^ availability observed after 4 months of exercise (Fig. 7a), may suggest that the long-term running regimen could slow down the impairment of SIRT1-related pathways that occurred during the transition from mature to middle age. In fact, NAD^+^ is known to be a critical co-factor for the deacetylating action of SIRT1 [63].

In summary, the 4-mo enforced running regimen strongly reduced the extent of oxidative and MG-dependent molecular damage, enhanced the removal of MG precursors, activated the aerobic energy catabolism and preserved the SIRT1-dependent neuroprotective pathway. To the best of our knowledge, this is the first experimental report dealing with a time-course analysis of the MG-, SIRT1- and redox/energy-related responses activated in the mammal hippocampal area by a late-life forced running program.

In this study, we unveiled another novel phenomenon. Indeed, the regular and moderate exercise program elicited a biphasic response in mouse brain hippocampi. We previously reported a non-monotonic response pattern triggered by a lately-initiated long-term exercise in mouse brain cortices [50]. In this study, we observed that 2 months of regular and moderate treadmill running strongly weakened the first line of enzymatic defense against hydrogen peroxide (Fig. 3) and decreased the pool of reduced GSH (Fig. 4) within the mouse hippocampal formation. Coherently, in 2-mo exercised mice we found a significant increase in the peroxidative damage (Fig. 2). Taken together, these results may suggest that, as seen in the cortex [50], the hippocampus undergoes significant redox imbalance during the first period of the exercise program. However, as several redox-based signalling pathways and physiological adaptations are activated by ROS and mild oxidative stress [16], [64], such initial imbalances might have an important role in preparing the cellular environment for the subsequent beneficial modifications we revealed after 4 months of running. Other parameters were significantly affected by the first period of the exercise program in a way that anticipated the effects of physiological aging. In fact, GAPDH activity and SIRT1 protein level were negatively regulated by 2 months of moderate running, while GLO1 expression resulted to be increased (Figs. 5a, 7b and 6, respectively). Together with the observed reduction in the amounts of NAD^+^ (Fig. 7a) and with the increasing trend relative to MG-modified protein levels (Fig. 6a), these findings seem to suggest that the first period of lately-initiated moderate exercise could facilitate the accumulation of cytotoxic and pro-oxidant dicarbonyl compounds (e.g., the MG), and this, in turn, could trigger an adaptive upregulation of the enzymatic machinery through which the MG is catabolized. Indeed, several stressors promoting MG accumulation are known to upregulate the expression or the activity of the main enzyme through which MG is catabolized, as a part of a metabolic adaptive response [65], [66]. This could also explain the decline of the reduced glutathione (Fig. 4), since catalytic amounts of this tripeptide are critically required for the GLO1-mediated removal of MG [67]. Again, such a complex pattern of metabolic and molecular changes may not be linked to a deleterious biochemical milieu; these modifications should have an important role in facilitating the hormetic adaptations we observed in the hippocampi of 4-mo exercised mice.

Biphasic responses to exercise have been already reported in muscles. Indeed, short-term exercise increases damage which in some studies reverses with longer exercise [15], [16], [43]–[46]. The fact that mouse hippocampus also responds to exercise as a function of the running duration is new. Supporting our previous work which was carried out on rodent brain cortices [50], this study strongly indicated that a long-term, forced but moderate running regimen initiated lately in adult life triggered a double-step effect on several crucial biomolecular networks and pathways within the mammal CNS.

In conclusion, our results suggested that the duration of exercise causes a profound shift in the response of the mouse hippocampus to regular running in a life-dependent fashion. Even when lately-initiated in mice unfamiliar with exercise, a long-term, forced and moderate treadmill running program elicited multiple beneficial adaptive responses, through the enhancement of the removal mechanism of MG precursors, the reduction of ROS- and MG-dependent damages, the activation of mitochondrial catabolism and the preservation of SIRT1-related neuroprotective pathways. However, these same cellular pathways were affected by the exercise regimen in a double-step fashion, maybe as a part of a complex regulatory process aimed at allowing the adaptive and hormetic responses triggered by long-term regular running.

Although many biomolecular details still remain to be studied, this research extends the knowledge on how a lately-initiated regular and moderate physical exercise could affect the earliest hallmarks of mouse brain senescence during the transition from adult to middle age.

In perspective, our data may help to develop lifestyle-based non-pharmacological interventions aimed at retarding brain senescence, even in individuals unfamiliar with physical activity.
